# The sample size effect in metallic glass deformation

**DOI:** 10.1038/s41598-020-67813-w

**Published:** 2020-07-01

**Authors:** Yannick Champion, Nicolas Thurieau

**Affiliations:** 1 Univ. Grenoble Alpes, CNRS, SIMaP, 38000 Grenoble, France; 2 IMSIA, CNRS, EDF, CEA, ENSTA Paris, Institut Polytechnique de Paris, 828, boulevard des Maréchaux, 91762 Palaiseau, France

**Keywords:** Materials science, Condensed-matter physics, Theory and computation

## Abstract

The sample size effect on deformation mode of glasses is one of the most misunderstood properties of this class of material. This effect is intriguing, since materials deemed macroscopically brittle become plastic at small size. We propose an explanation of this phenomenon for metallic glasses. A thermodynamic description of the local rearrangement zones activated under an applied stress is proposed. Using the Poisson distribution to describe the statistics of these zones and the statistical physics to associate entropy, we define a critical sample size for the change in the deformation mode. Predictions are in agreement with experimental observations and reveal hidden structural parameters describing the glassy state.

## Introduction

As is well known for millenaries, oxide glass flows at high temperature, which allows forming bottles, window glasses and so on, but are extremely brittle below the so-called glass transition temperature. Less common materials, metallic glasses behave in the same way at the macroscopic level. Of course, fundamental mechanisms for absence of macroscopic plastic deformation are distinct between oxides and metals due to their difference in atomic bonds. However, in both these cases the brittleness stems from a same effect that is stress localization leading to formation of a crack in oxides and a shear band in metallic glasses. It is then very intriguing to observe homogenous plastic deformation when glass sample is getting sufficiently small. The first evidence was reported by Taylor in 1949 using indentation on a borosilicate optical glass^1^. Since, mechanical tests on micro pillars reveal plasticity for diameter of sample of few micrometers^2^. For metallic glasses, the first evidence was reported by Volkert and collaborators on a PdSi alloy nano-pillars with diameter of 440 nm^3^. So far, an explanation has been based on a necessary critical volume for crack or shear band formation and propagation^3,4^. With analogy to the Griffith criterion, the critical size is estimated from the balance between elastic energy stored under the applied stress $\sigma$ and the crack or shear band energy, $\Delta \propto\Gamma E/{\sigma }^{2}$ where $\Gamma$ is the energy per unit area of crack or shear band and $E$ is the Young modulus. In addition to change in a deformation mode from brittle to ductile, increase of strength with decreasing size is observed. This has been associated to shear bands inhibition^5^ or explained by a shear banding mediated process which takes into account the stochasticity observed at the critical size^6^. Origin and mechanism for the change in the deformation mode from brittle to ductile in glasses is still debating. In their work, Guo and collaborators^7^ observed in situ at the microscopic scale, using transmission electron microscopy, the homogenous deformation of a metallic glass followed by instability leading to necking similar to deformation in crystals. Evidence of shear band inhibition was interpreted through several angles. With the “Griffith criterion”, they proposed the interesting aspect of necessary distance for shear band to reach a mature stage. In addition, the authors emphasized the fact that the sample size is most likely below the range of shear band spacing. They suggest that the sample volume is less likely to contain “fertile” sites for the initiation of shear bands (those favorable to form shear transformation zones).

The mechanical approach is necessarily based on shear band properties. We suggest an alternative approach starting on the idea that the glass flows at a critical size as it does at the glass transition temperature. Hence, a “sample size—temperature” equivalence is envisaged similar to the “time–temperature” equivalence in thermally activated process. We shall see that this is more specifically a “size—glass transition temperature” equivalence.

The glass transition is understood as the temperature below which elementary species mobility is too low to observe relaxation at the time scale of experiment. In their initial statistical mechanics theory, Gibbs and DiMarzio, showed that the increase in relaxation time at the glass transition is related to a dramatic decrease towards very small value of the configurational entropy^8^. Later on, Gibbs and Adam introduced the configurational entropy in description of the viscosity^9^, what Angell discussed in term of entropy excess of liquid to that of the crystal in the supercooled domain^10^. From that, an interpretation of the phenomenon would be that an increase in specific configurational entropy happens with decreasing sample size. Homogenous deformation should be then allowed when the value at the glass transition is reached. A change in entropy with size has not been considered yet, but was the number of energy minima in the potential energy landscape (PEL) description of glass^11,12^. The PEL is appropriate for modelling the structural local rearrangement under an applied stress and the variation in minima of the energy density supports well an evolution of configuration with size.

## Model and experimental evidence

**Figure 1:**
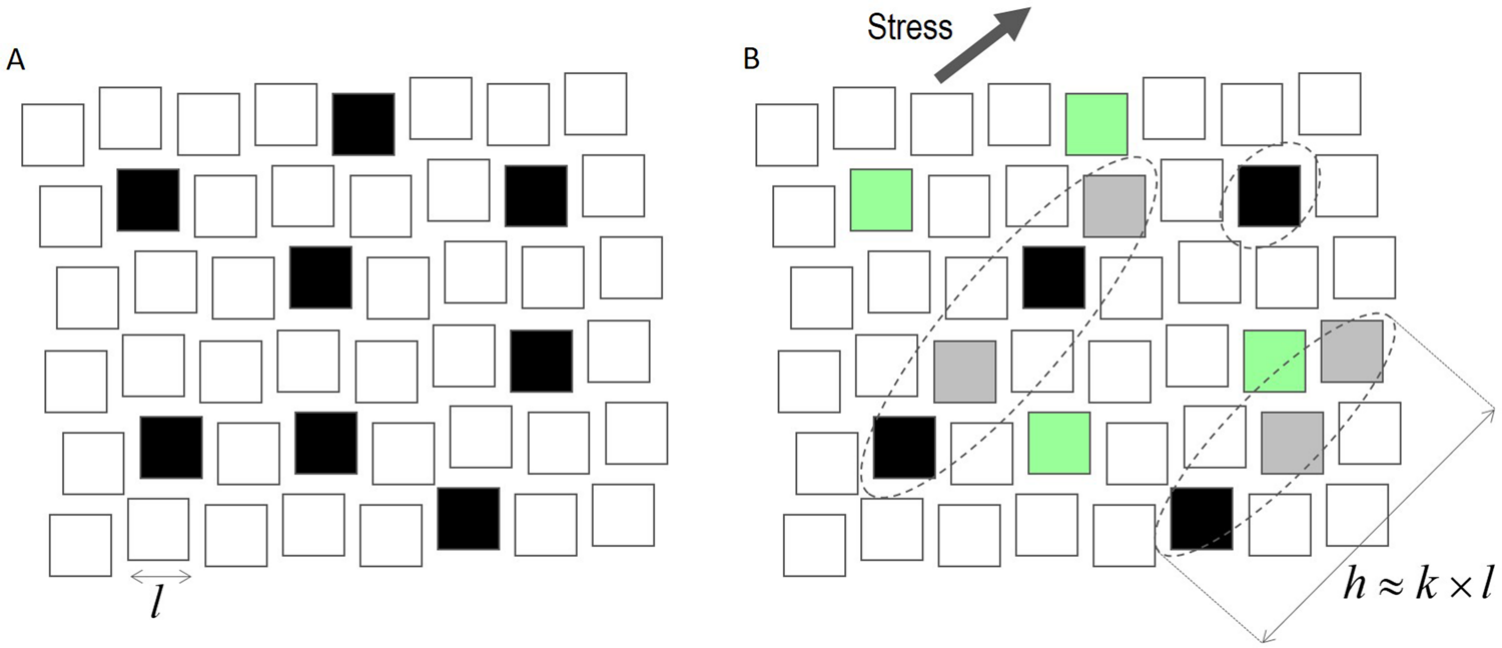
2D scheme of the glass description in a PEL approach. (**A**) The glass is divided in “clusters” with same size, formed in average of the same number but different atoms. Clusters are distributed over the PEL, where some (black) are in favorable energy configuration with respect to an external solicitation. (**B**) Orientation of the external stress solicitation produces selection of clusters (black) favorably oriented. Though energetically favorable, green clusters are not favorably oriented with respect to the applied stress. In the complex PEL gradient, clusters (grey) nearby selected black clusters, energetically and favorably oriented can be triggered participating in the rearrangement in the stress direction.

The glass is discretized in non-periodic small regions, we call “clusters” of size $l$, having various potential energy in the PEL distribution. Figure 1A, is a 2D scheme of a glass in such description, where black clusters are in favorable energy configuration for rearrangement under an external solicitation. A stress is characterized by its spatial orientation and breaks the average spherical symmetry of the glass. It results that only clusters favorably oriented with respect to the applied stress are selected and the resulting atomic displacements are in a close direction to the stress orientation (Fig. 1B). The PEL is heterogeneous and locally non-isotropic^13^. Then, activation of a favorable cluster, (black in Fig. 1B) may trigger atomic rearrangement over many $k$ successive clusters nearby (Fig. 1B). We call ligament of size $h\approx k\times l$ , the successive clusters (black and grey in Fig. 1B) producing the total displacement $h$. The total number of clusters is very large (${n}_{c}\gg 1$) and the probability to find a ligament is very small ($p\ll 1$). Such rare event configuration is described by a Poisson distribution which gives the probability ${P}_{k}={\lambda }^{k}{e}^{-\lambda }/k!$, of finding ligaments formed of $k$ successive clusters, where $\lambda =p\times {n}_{c}$ is the average number of clusters giving the most expected ligament size $\overline{h}=\lambda \times l$ .

This model description was tested by comparison with experimental data. At first, the predicted displacements were naturally associated to serrations (pop-in) events observed in nano-indentation or nano-pillar compression tests. The mechanical test is probing the local structure of the glass by observing in the displacement extent (serration size) the capacity for atomic rearrangement. It is well known that the serrations events are strain rate dependent^14^, which would mean that the serrations size distribution is not unique. However, it is emphasized that the most faithful structural description obtained from the mechanical probing, necessarily required that the time scale of the experiment is lower than the timescale of the atomic rearrangement. To an experimental point of view, it is then obvious that such experiment must be carried out in quasisatic condition that is at the lower strain rate as possible.

**Figure 2:**
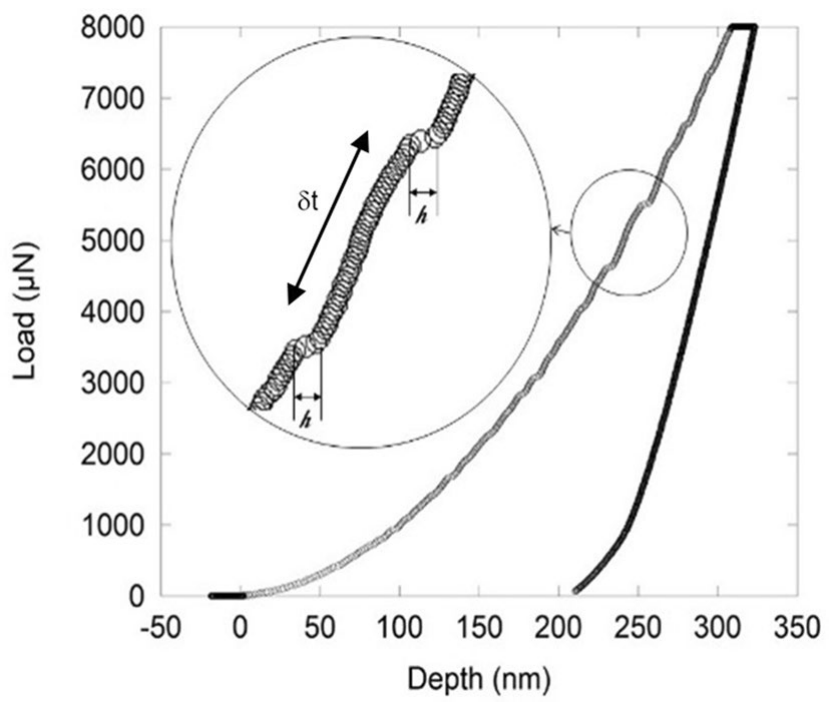
Nano-indentation curve showing serrations on the loading branch, also shown in the inset with the serration size, $\mathrm{h}$ and the waiting time between successive serrations $\updelta \mathrm{t}$.

About 7,980 serrations were measured from 320 nano-indentations (Fig. 2) performed on a Mg based metallic glass^15^ (see supplementary materials and methods).

**Figure 3:**
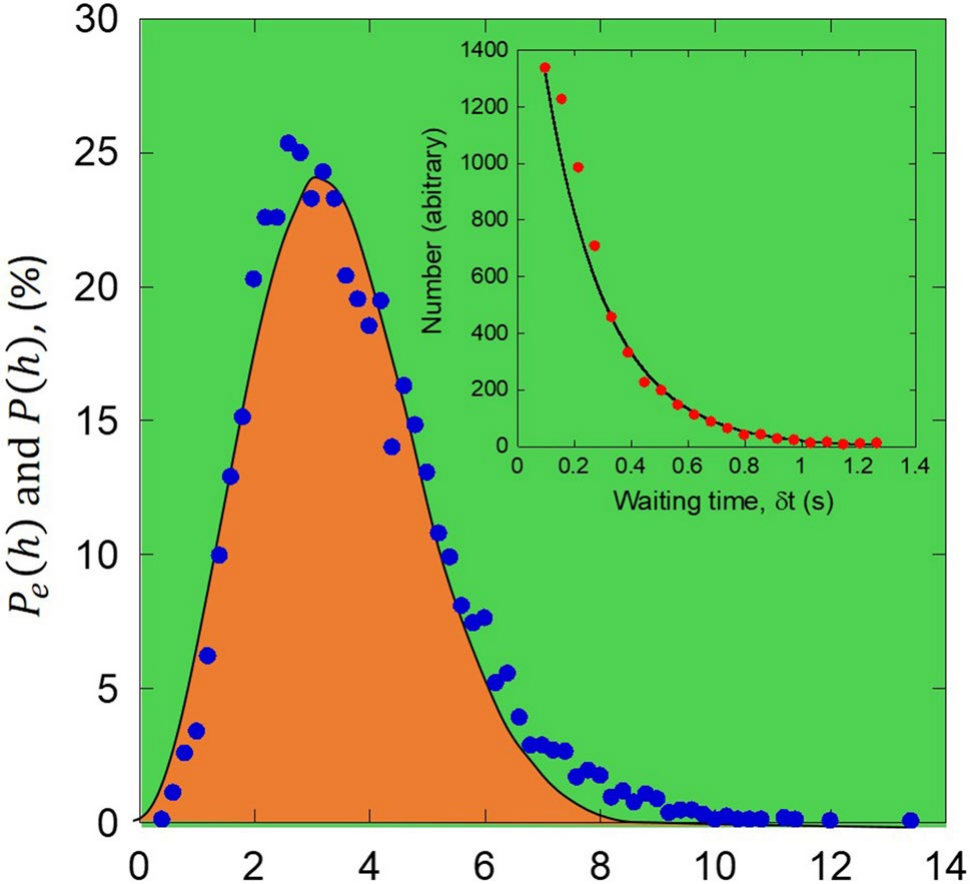
Normalized experimental distribution of serration sizes ${\mathrm{P}}_{\mathrm{e}}(\mathrm{h})$ (blue dots) compared with the Poisson distribution $\mathrm{P}(\mathrm{h})$. Distribution of waiting time, $\updelta \mathrm{t}$ between successive serrations is shown in the inset.

The normalized experimental distribution ${P}_{e}(h)$ is plotted in the Fig. 3 and compared to a normalized Poisson distribution $P(h)$ with the fitting parameters: $\overline{h}$=3.47 ± 0.03 nm, $l$=0.64 ± 0.03 nm, which gives $\lambda \approx 5$. The waiting times, $\delta t$ distribution (inset of Fig. 3) is also consistent with the Poisson statistic and verifies $P\left(\delta t\right)= {Ae}^{-\lambda \delta t}$. It was reported that the activation volume controlling the shear band formation in this Mg glass is of the order of 3 atoms^16^. Similar value was reported by Schall and collaborators^17^ and Ju and collaborators^18^ for different materials. The present statistical analysis is consistent with that result, considering that an elementary displacement of the order of $l$, which is about two interatomic distances, needs a rearrangement of at least 3 atoms. It is satisfactory to find analogy between cluster size used for the discretization of the glass and the activation volume controlling shear band formation having a physical meaning.

In a statistical physics approach, as proposed by Gibbs and Adam^9^, the probability to find a ligament formed of $k$ clusters is ${P}_{k}={g}_{k}{e}^{\frac{-{u}_{k}}{{k}_{b}T}}/Z$. ${u}_{k}$ is the energy of a ligament of $k$ clusters in the PEL, ${g}_{k}$ is a degeneracy factor and $Z$ is the partition function. If we arbitrarily set for the most probable ligament of $\lambda$ clusters, ${g}_{\lambda }$ =1, then combining with the Poisson distribution and using definition of the free energy, $f=-{k}_{b}TlnZ={u}_{\lambda }-{k}_{b}Tln\left(\frac{\lambda !}{{{\lambda }^{\lambda }e}^{-\lambda }}\right)$. ${k}_{b}$ is the Boltzmann constant and $T$ is the absolute temperature. Energy of the ligament is unknown but we derive a simple apparent entropy per ligament depending only on $\lambda$, $s={k}_{b}ln\left(\frac{\lambda !}{{\lambda }^{\lambda }{e}^{-\lambda }}\right)$.

## The size effect

Among the simplicity of the result, the complexion $\Omega =\frac{\lambda !}{{{\lambda }^{\lambda }e}^{-\lambda }}$ brings interesting perspectives. The Permutations ($\lambda !$) and the combinations (${\lambda }^{\lambda }$) of the $\lambda$ clusters forming the most probable ligament are identified. It seems that ligaments formed of $\lambda$ clusters are only considered and then a large part the glass is missing. However, rewriting ${e}^{-\lambda }={\mathrm{lim}}_{{n}_{c}\to \infty }{\left(1-\frac{\lambda }{{n}_{c}}\right)}^{{n}_{c}}$ reveals the combinations of the fraction of the ${n}_{c}-\lambda$ , other clusters, indicating that the overall sample is well considered in the complexion. Further, identifying that ${{{\mathrm{l}\mathrm{i}\mathrm{m}}_{{n}_{c}\to \infty }\left(1-\frac{\lambda }{{n}_{c}}\right)}^{{n}_{c}}\approx {\mathrm{l}\mathrm{i}\mathrm{m}}_{n\to \infty }\left(1-\frac{\lambda }{n}\right)}^{n}$ , $n$ is the total number of ligaments, inserting in the entropy expression and after Taylor series development, a size dependent of entropy is derived:

1 $$s={k}_{b}ln\left(\frac{\lambda !}{{\lambda }^{\lambda }{e}^{-\lambda }}\right)+{k}_{b}\frac{{\lambda }^{2}}{2n}$$

Reducing $n$ means that the statistic is changing from Poisson to binomial, a well-known property for these distributions. In other words, the way events are “drawn” changes with the reduction of the sample size. From this result and considering the Gibbs and Adam analysis^9^, we define the “sample size—temperature” equivalence writing that the entropy of an infinite sample at the glass transition temperature is equal to the entropy of the small size specimen at room temperature. This simple equality needs however, some special care. Entropy from room temperature to glass transition is calculated numerically with heat capacity ${c}_{p}$ measured for many metallic glasses (Table 1). It is emphasized that entropy of the small size sample is calculated with data obtained from the mechanical testing, then considering only the ligament probed by the mechanical solicitation in its specific direction. Consequently, the entropy from room to glass temperature must be rescaled by the ligaments fraction probed in the mechanical testing. Heat capacity ${c}_{p}$ is rescaled in number of ligaments (or mole of ligaments), that is by $\sim 3\lambda$, assuming cluster formed of about 3 atoms. The balance between the entropy of the small size specimen and the entropy of infinite specimen at the glass transition writes, with $\Omega$ the atomic volume and $\frac{{n}_{l}}{n}$ the fraction of ligaments probed:

**Table 1:** Comparison between observed and calculated critical size $\Delta$ for various metallic glass alloys. Structural parameters, glass transition temperature and entropy are indicated.

**Glass**	**Test**	${\varvec{\lambda}}$	${\varvec{\Psi}}$**(nm**^**-3**^**)**	${\int }_{{{\varvec{T}}}_{{\varvec{R}}{\varvec{T}}}}^{{{\varvec{T}}}_{{\varvec{g}}}}{{\varvec{c}}}_{{\varvec{p}}}\frac{{\varvec{d}}{\varvec{T}}}{{\varvec{T}}}$**(J.mol**^**-1**^**.K**^**-1**^**)**	**Tg (K)**	**Size (nm) observed**	${\varvec{\Delta}}$** (nm) calculated**
Pd_77_Si_23_	Pillar^3^	20	1 × 10^–8^	20 from^25^ Pd_80_Si_20_	480	440	500
Mg_65_Cu_12.5_Ni_12.5_(Ce_0.75_La_0.25_)_10_	Indent this work	5	1 × 10^–8^	10 from^19^ Mg_65_Cu_25_Y_10_	425	< 1,000 from^20^ Mg_65_Cu_25_Gd_10_	410
Au_49_Ag_5.5_Pd_2.3_Cu_26.9_Si_16.3_	Pillar^26^	7	1.6 × 10^–8^	10.5 from^27^ Au_53.2_Pb_27.5_Sb_19.2_	400	< 1,000	400
Cu_47_Ti_33_Zr_11_Ni_6_Sn_2_Si_1_	Pillar^28^	9	8.5 × 10^–7^	30 from^29^ Cu_47_Ti_34_Zr_11_Ni_8_	673	70	80
Zr_47_Cu_46_Al_7_	Pillar^30^	7	1.6 × 10^–6^	30 from^31^ Zr_41.2_Ti_13.8_Cu_12.5_Ni_10_Be_22.5_	675	< 100	60
Zr_41_Ti_14_Cu_12.5_Ni_10_Be_22.5_	Pillar^32^	9	2.4 × 10^–6^	30 from^31^ Zr_41.2_Ti_13.8_Cu_12.5_Ni_10_Be_22.5_	630	< 75	55

2 $${k}_{b}ln\left(\frac{\lambda !}{{\lambda }^{\lambda }{e}^{-\lambda }}\right)+{k}_{b}\frac{{\lambda }^{2}}{2n}={k}_{b}ln\left(\frac{\lambda !}{{\lambda }^{\lambda }{e}^{-\lambda }}\right)+3\lambda\Omega \frac{{n}_{l}}{n}{\int }_{{T}_{RT}}^{{T}_{g}}{c}_{p}\frac{dT}{T}$$

The volume of the specimen is $V=n3\lambda\Omega$ and the volume density of ligament is $\Psi =\frac{{n}_{l}}{V}=\frac{{n}_{l}}{n3\lambda\Omega }$.

After combination, the critical size for the transition in the specimen size effect is obtained:

3 $$\Delta =\sqrt[3]{\frac{{k}_{b}\lambda }{6\Psi {\int }_{{T}_{RT}}^{{T}_{g}}{c}_{p}\frac{dT}{T}}}$$

$\lambda$ and $\Psi$ are structural features of the glass determined from experiments and characterizing the deformation dynamics.

For the Mg based metallic glass used in our experimental work, $\lambda =5$ is determined from a robust statistical distribution. The ligaments density is estimated of 10^–8^ nm^−3^, assuming a hemispheric zone, $v\approx 60{h}_{max}^{3}$, where ${h}_{max}$ is the maximum indent depth. An entropy from room temperature to glass transition was numerically evaluated from^19^ of about 10 J.mol^-1^ k^-1^. Then the critical size for the transition from brittle to ductile is calculated of about 400 nm, consistent with observations by Lee and collaborators on pillars with diameter smaller than 1,000 nm^20^.

## Discussion and concluding remarks

The approach was applied for various metallic glasses tested on nanopillars (Table 1). The volume of pillar, impacted during deformation is $v\approx \epsilon V$, where $\epsilon$ is the strain and $V$ is the pillar volume. The $\lambda$ value is estimated from the serration which appears the most (the much probable) in the deformation curves and elementary cluster formed of 3 atoms is assumed. The Table 1 shows rather good estimation of the critical size when comparing experimental observations and the calculated values, $\Delta$.

To come back on initial assumption of a “sample size—${T}_{g}$” equivalence, one relies on the relation of the critical size (3). Observing that the ${c}_{p}$ variation with temperature are little for the various glasses (${c}_{p}$ is from 25 to 90 J.K^-1^.mol^-1^), one derives the size-temperature dependence, from the first terms of a Taylor development:

4 $$\Delta \propto \sqrt[3]{\frac{\lambda }{\Psi }\frac{1}{{C}_{p}({T}_{g}/{T}_{RT}-1)}}$$

**Figure 4:**
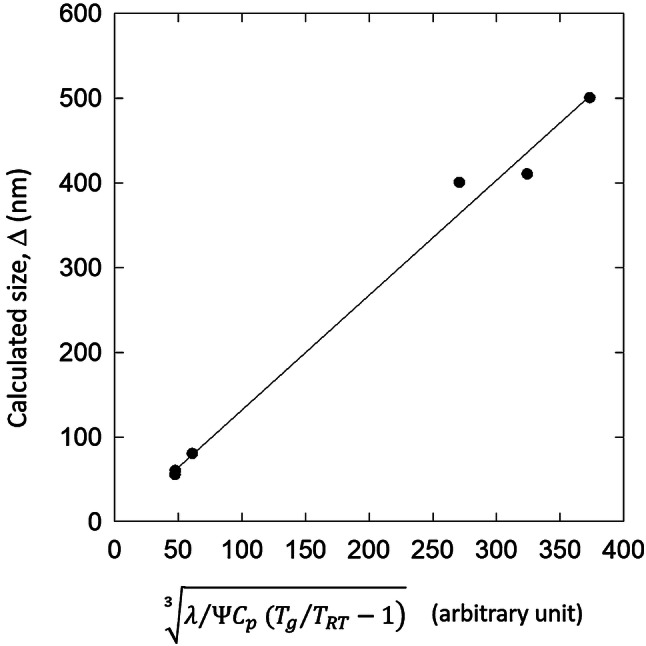
Dependence of the critical size $\Delta$ with the glass transition temperature ${\mathrm{T}}_{\mathrm{g}}$.

The relation demonstrate the starting point of the approach with the size ($\Delta$)—temperature (${T}_{g}$) or entropy (${C}_{p}, {T}_{g}$) dependence (Fig. 4). The relation (4) also reminds that this equivalence is done between two different property-dependent values, a mechanical one ($\Delta$) and thermal one (${T}_{g}$) that is why structural parameter, $\lambda ,\Psi$ are necessary to make compatibility between the two. The relation (4) predicts that the critical size is as small as Tg and $\Psi$ are large, which is the case for Zr, and Cu based alloys, compared to Pd, Mg, Au-based alloys. $\lambda$ values are similar except for PdSi glass (Table 1). In this work, the reference temperature is the room temperature; it is interesting to notice that infinite critical size is well predicted for reference temperature of ${T}_{g}$ and that critical size $\Delta \to 0$ when $\mathrm{T}\to 0$. This donnot consider variations of value $\lambda$ and $\Psi$ with temperature, which is most likely the case.

A dominant parameter impacting the critical size $\Delta$, is the volume density of ligaments as observed from the data in the Table 1. $\Psi$ is the concentration of the local zones in the glass where rearrangement is able to occur under stress. One of these zones will evolve forming shear band. It is commonly argued that multiplying shear bands would be much favorable for plasticity while our results would indicate the contrary. The critical size is as large as $\Psi$ is small. In other words, the glass would be as robust against brittleness as it is poor in easy rearranging zones where softening happens under stress. This was called “fertile” zones by Guo and collaborators^7^. The argument support well the evidence of large critical size observed for oxide glasses which are probably more “structurally perfect” compared to metallic glasses. This should be examined under the angle of difference in the PEL between oxide and metallic glasses.

In the deformation process, $\lambda$ and $\Psi$ are novel parameters for describing the glass structure. It has been shown that metallic glass is formed of a distribution of clusters having varied deviations from a perfect icosahedron^21^. The local structure was earlier described by a distribution of atoms and free volume, which is convenient in particular for modeling mechanical behavior as developed by Spaepen^22^ and Argon^23^. An alternative to direct or mean field atomic structure is the PEL^11^ where atoms are omitted and the properties related to local variation of the system energy. This was used for the modelling glass rheology^24^. Our approach suggests that the glass can be described by the unique average value $\lambda$ and the density of ligaments $\Psi$. The first is corresponding to elementary translation in the glass as Burgers vector is in crystals. The second is the density of local “defects” involved in the deformation process analogous to dislocations density in crystals though having different properties.

## Supplementary information

Supplementary file1 (DOCX 13 kb)
