# Supplementary material for: The sample size effect in metallic glass deformation
Source: Sci Rep. 2020 Jul 1;10:10801. doi: 10.1038/s41598-020-67813-w (PMC7330049; doi:10.1038/s41598-020-67813-w)
Supplement: Supplementary file 1 — Supplementary file1 (DOCX 13 kb) [file 41598_2020_67813_MOESM1_ESM.docx]

**The sample size effect in metallic glass deformation**

Yannick Champion^1*^, Nicolas Thurieau^2^.

^1^Univ. Grenoble Alpes, CNRS, *SIMaP*, 38000 Grenoble, France.

^2^ENSTA Paris - Institut Polytechnique de Paris, 828, boulevard des Maréchaux 91120 Palaiseau, France.

*Correspondence to: [yannick.champion@grenoble-inp.fr](mailto:yannick.champion@grenoble-inp.fr).

**Supplementary material**

**Preparation of the metallic glass and nano-indentation experiments**

The experiments reported in this article were conducted on a Mg_65_Cu_12.5_Ni_12.5_MM_10_ alloy. Electromagnetic melting of Mg, Cu, Ni and mischmetal (Ce_75_La_25_) was carried out in a cold crucible under controlled atmosphere. The metallic glass was then produced by twin roll casting of the liquid alloy in He atmosphere in the form of a sheet with a thickness of about 400 µm, a width of about 3 cm and a length of about 30 cm. The amorphous nature was checked by X-ray diffraction. Nanoindentation was performed with a TI 950 Tribo Indenter from Hysitron equipped with a Berkovich tip (threefold symmetry pyramid). For each measurement, the load was applied at a loading rate of 4mN.s^-1^ up to the maximum load of 8000 μN. Strain rate, $\dot{\varepsilon}{=dh}/{hdt}$ ( $h$ is the indent depth and $t$ is the time) is extremely high at the onset of the deformation and thus serrations are too small due to strain rate effect, to be detected. After about 100 nm indentation depth, the strain rate is nearly constant at a sufficiently low value of about 1 to 0.3 s^-1^ (nearly quasistic conditions). Serrations size and waiting times between two successive serrations were automatically detected and measured. For that reason, a tailor-made automated procedure was used, based on slope discontinuity along the loading part of the indentation curves with a resolution in size (or depth displacement) better than 1 nm. 7980 serrations were measured from 320 indentation curves. This very large dataset enables relevant and rigorous statistical analysis, since the probability for observing large and thus very rare events is sufficiently high.
